# Cell sorting reveals few novel prokaryote and photosynthetic picoeukaryote associations in the oligotrophic ocean

**DOI:** 10.1111/1462-2920.15351

**Published:** 2020-12-19

**Authors:** Hanna Farnelid, Kendra Turk‐Kubo, Jonathan P. Zehr

**Affiliations:** ^1^ Ocean Sciences Department University of California Santa Cruz CA USA; ^2^ Centre for Ecology and Evolution in Microbial Model Systems (EEMiS) Linnaeus University Kalmar Sweden

## Abstract

Close associations between single‐celled marine organisms can have a central role in biogeochemical processes and are of great interest for understanding the evolution of organisms. The global significance of such associations raises the question of whether unidentified associations are yet to be discovered. In this study, fluorescence‐activated cell sorted photosynthetic picoeukayote (PPE) populations and single cells were analysed by sequencing of 16S rRNA genes in the oligotrophic North Pacific Subtropical Gyre. Samples were collected during two cruises, spanning depths near the deep chlorophyll maximum, where the abundance of PPEs was highest. The association between the widespread and significant nitrogen (N_2_)‐fixing cyanobacterium, UCYN‐A and its prymnesiophyte host was prevalent in both population and single‐cell sorts. Several bacterial sequences, affiliating with previously described symbiotic taxa were detected but their detection was rare and not well replicated, precluding identification of novel tightly linked species‐specific associations. Similarly, no enrichment of dominant seawater taxa such as *Prochlorococcus*, SAR11 or *Synechococcus* was observed suggesting that these were not systematically ingested by the PPE in this study. The results indicate that apart from the UCYN‐A symbiosis, similar tight species‐specific associations with PPEs are unusual in the oligotrophic ocean.

## Introduction

Associations between marine microbes are considered important drivers for shaping microbial communities and ecological processes (Azam and Malfatti, 2007; Lima‐Mendez *et al*., 2015; Worden *et al*., 2015). Key microbial associations could be identified as recurrent interactions, or close physical connections (attached or intracellular) in the form of symbiosis (Thompson and Zehr, 2013; Amin *et al*., 2015; Cooper and Smith, 2015) but may also include parasitism and association through grazing. In oligotrophic oceans, associations between bacteria and phytoplankton cells involving exchanges of nutrients, vitamins and/or metabolites may provide ecological advantages for the associated cells compared with their free‐living counterparts (Amin *et al*., 2012; Arandia‐Gorostidi *et al*., 2017). Associations between bacteria and phytoplankton have been frequently described and are considered widespread. They can be intracellular or extracellular ranging from loose to very tight physical interactions where cells have co‐evolved (Malfatti and Azam, 2009; Seymour *et al*., 2017). Examples of intracellular associations are intracellular symbionts or ingestion of a prokaryotic prey by mixotrophic cells. Despite of their importance, their generally low relative abundance within the microbial community makes them challenging to identify and study (Foster and Zehr, 2019). Consequently, there may be multiple key associations yet to be discovered and the metabolic interactions between the organisms in most known associations remain poorly characterized.

Marine photosynthetic picoplankton (≤2–3 μm in diameter) include the picocyanobacterial genera *Prochlorococcus* and *Synechococcus*, as well as photosynthetic picoeukaryotes (PPEs). Because of their high surface area to volume ratios, picoplankton are successful in low nutrient areas reaching high abundances (i.e. 10^2^ to 10^6^ cells ml^−1^) and contribute significantly to global autotrophic carbon biomass and primary production (Worden *et al*., 2004; Buitenhuis *et al*., 2012; Flombaum *et al*., 2013). Although less abundant than the picocyanobacteria, PPEs have been shown to be equally important in terms of both biomass and primary production (Worden *et al*., 2004; Jardillier *et al*., 2010). The PPEs are a taxonomically diverse group including representatives of the phyla Chlorophyta, Haptophyta, Cryptophyta and Heterokontophyta (Vaulot *et al*., 2008; Massana, 2011). Despite recent advances in characterizing PPEs using molecular techniques (Cuvelier *et al*., 2010; Worden *et al*., 2011; Keeling *et al*., 2014), many of the dominating taxa remain uncultured which limits information about their morphology and physiology.

The symbiosis between the unicellular nitrogen (N_2_)‐fixing cyanobacterium UCYN‐A and its PPE (prymnesiophyte) host is an example of an intimate association which is based on the exchange of N and C between the cyanobacterium and the host (Thompson *et al*., 2012; Krupke *et al*., 2013). This association is widely distributed in the oligotrophic ocean and UCYN‐A is a significant contributor to global N_2_ fixation (Moisander *et al*., 2010; Farnelid *et al*., 2016a; Martínez‐Pérez *et al*., 2016). Given the success of this association, it can be hypothesized that similar associations may be prevalent in the marine environment (Zehr, 2015). For example, it has been suggested that heterotrophic N_2_‐fixers may facilitate N_2_ fixation in the oligotrophic ocean by living in close association with phytoplankton cells (Riemann *et al*., 2010; Bombar *et al*., 2013). Thus, these types of associations are known to mediate metabolic processes that are key to biogeochemical cycling in the oligotrophic oceans, and there may be additional important associations that have thus far eluded detection.

Studies have suggested that PPEs may be ecologically important bacterivores (Zubkov and Tarran, 2008; Hartmann *et al*., 2012, 2013) thus playing important roles both as primary producers and as grazers of prokaryotes in marine waters. Using a combination of fluorescence‐activated cell sorting (FACS) and dual tyramide signal amplification fluorescence in situ hybridization, Hartmann *et al*. (2013) observed both attached and ingested bacterioplankton cells associated with PPEs in the oligotrophic Atlantic Ocean. Similarly, methodologies including FACS followed by molecular analyses have suggested close ecological associations between bacterioplankton and PPEs in coastal environments (Brown *et al*., 2020; Martinez‐Garcia *et al*., 2012; Farnelid *et al*., 2016b). Such single‐cell approaches, albeit limited to associations that are not disrupted by FACS, allow for the identification of tightly linked species‐specific associations and have the potential for the discovery of previously unknown associations.

The purpose of this study was to identify and investigate close associations between bacterioplankton and PPEs in the oligotrophic North Pacific Subtropical Gyre (NPSG). To obtain these populations, which were minor components of the microbial community, raw seawater samples from depths around the deep chlorophyll max (DCM) were collected during two cruises from different seasons (February and July 2015). PPE populations and single PPE cells were sorted using FACS and co‐sorted bacteria were identified using PCR amplification of the 16S rRNA gene and Illumina MiSeq sequencing. Bulk community samples were analysed to evaluate if there was an enrichment of certain bacterial taxa among the sorted cells. In total, 23 populations of *Synechococcus*, *Prochlorococcus* or PPE with 1000 sorted cells each in triplicates and 388 single amplified genomes of which 78 produced data were screened. To our knowledge, this study is the most extensive analysis to date to identify novel species‐specific associations between bacterioplankton and PPEs, which may be of large ecological significance.

## Results

**Fig. 1:**
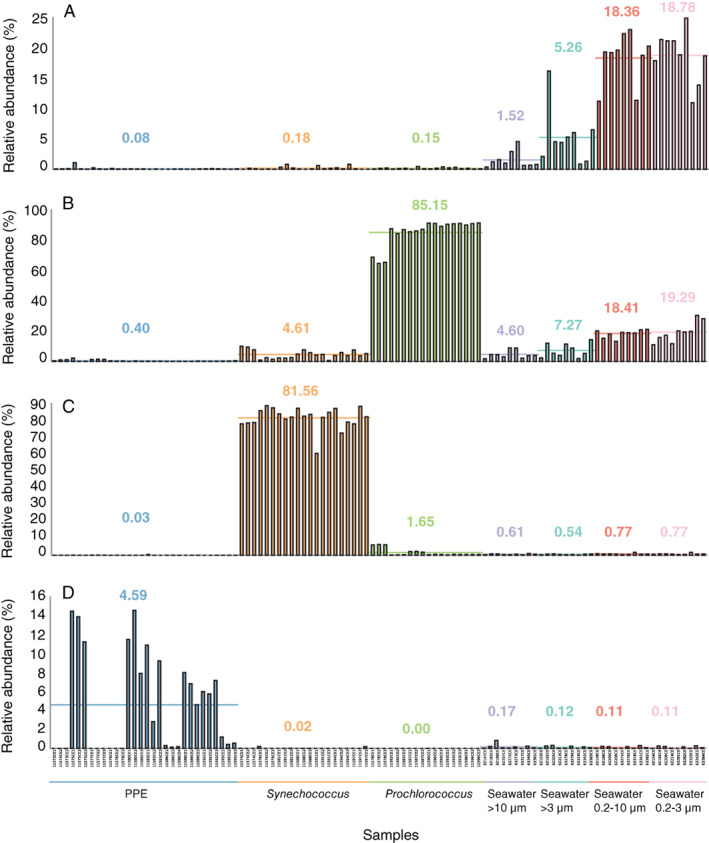
Bar graphs of the relative abundance (%) of sequences affiliating with A. *Pelagibacter*, B. *Prochlorococcus*, C. *Synechococcus* and D. UCYN‐A (denovo223459) in each sample group according to Table 1 (PPE sorts, *Synechococcus* sorts, *Prochlorococcus* sorts, Seawater >10 μm, Seawater >3 μm, Seawater 0.2–10 μm, and Seawater 0.2–3 μm). The mean relative abundance is indicated by horizontal lines and written in colours representing each sample group.

Sampling depths between 75 and 140 m were chosen to coincide with the highest abundances of PPEs (Table 1, Supplementary Table S1, Supplementary Figs 1 and 2). At Station ALOHA, during the HOT269 cruise, the abundances of PPEs ranged from 1.5 × 10^6^ to 2.0 × 10^6^ cells L^−1^ with the higher abundance of PPE cells found close to the DCM (between 120 and 130 m). During the HL2B cruise, Lagrangian sampling was performed in an anticyclonic eddy to the Northeast of Station ALOHA (Wilson *et al*., 2017; Farnelid *et al*., 2019). The abundances of PPEs ranged from 0.6 × 10^6^ to 2.6 × 10^6^ cells L^−1^ with the maximum abundance at 125 m. As typical in the NPSG, oligotrophic free‐living bacteria such as the alphaproteobacterium SAR11 (*Pelagibacter ubique*) and *Prochlorococcus* were dominant in the seawater samples with an average relative abundance of up to 18.8% and 19.2% respectively (Fig. 1 and Supplementary Table S2).

**Table 1:** List of sorted samples analysed.

Date collected	Cruise	PPE sort sample ID	Depth (m)	Syn (cells)	Pro (cells)	PPE (cells)	SAGs	Seawater (Y)
2015_02_24	HOT269	11586	140	1000	1000	1000	–	Y
2015_02_25	HOT269	11589	100	100	1000	1000	–	Y
2015_02_25	HOT269	11592	75	100	1000	1000	–	Y
2015_02_25	HOT269	11595	125	100	1000	1000	–	Y
2015_07_26	HL2B	11575	75	1000	–	1000	–	Y
2015_07_26	HL2B	11573	100	1000	–	1000	–	Y
2015_07_28	HL2B	–	75	–	–	–	2	–
2015_07_29	HL2B	–	75	–	–	–	30	–
2015_07_29	HL2B	–	75	–	–	–	17	–
2015_07_29	HL2B	–	75	–	–	–	29	–
2015_07_30	HL2B	11579	100	–	–	1000	–	–
2015_07_30	HL2B	11577	125	–	1000	1000	–	Y
2015_07_31	HL2B	11580	75	1000	1000	1000	–	Y
2015_08_01	HL2B	11583	100	–	–	1000	–	Y

For population sorts, each sample was analysed in triplicates for each sorted group. *Synechococcus*, Syn; *Prochlorococcus*, Pro; Photosynthetic picoeukaryotes, PPE; SAGs, number of single amplified genomes which passed quality controls; Seawater (Y) indicates when size‐fractionated seawater samples (>10, >3, 0.2–10 and 0.2–3 μm) were sampled; −, not sampled.

### Composition of the PPE community based on chloroplast sequences

**Fig. 2:**
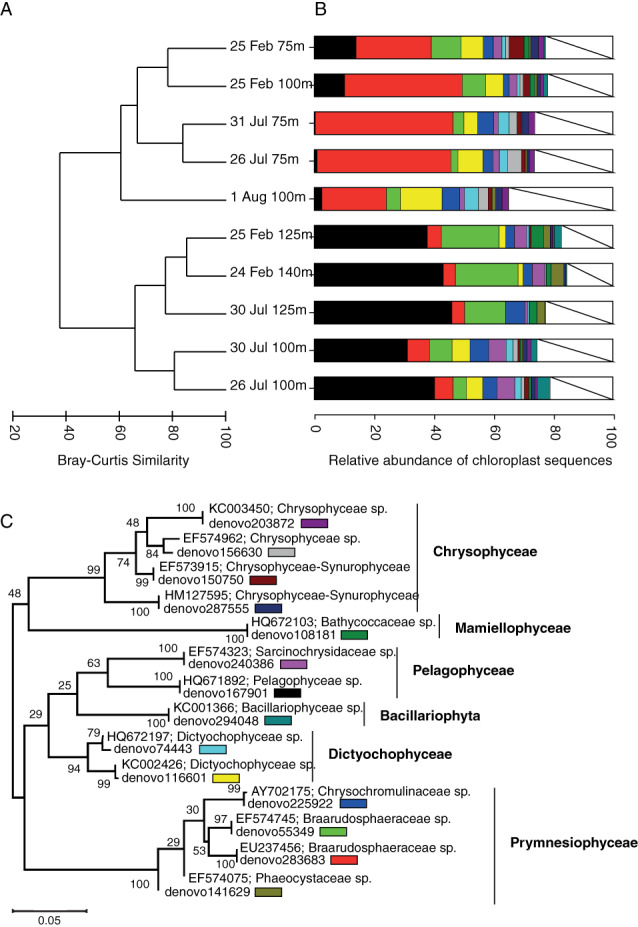
A. Bray–Curtis similarity dendrogram showing the similarity of the sorted PPE populations based on average triplicate sorts (1000 PPE cells each) from each sample, including all chloroplast sequences. B. Stacked bar graph of the average relative abundance of the top chloroplast OTUs (>1% of total chloroplast sequences) for each sample based on triplicate sorts. C. Maximum likelihood inferred phylogenetic tree of the top chloroplast OTUs and their nearest relatives with GenBank accession numbers and classification according to the PhytoRef database. Bootstrap values (1000 iterations) are indicated next to the branches. Colours next to the OTUs indicate the colour as shown in Fig. 1B, white colour indicates the sum of OTUs of <1% relative abundance.

A large proportion of the sequences from the sorted PPE populations were from chloroplasts (ranging from 62% to 99% average relative abundance of population libraries; Supplementary Table S3). The chloroplast compositions of the samples were separated into two clusters of samples based on Bray–Curtis similarity (Fig. 2A). One of the clusters, consisting of sorts from 75 and 100 m from both cruises, was dominated (22%–46%) by an operational taxonomic unit (OTU) (denovo283683) related to the chloroplast sequence of the prymnesiophyte *Braarudosphaera bigelowii* (100% identity; Accession number AB847986), which has been identified as the picoeukaryote partner cell of UCYN‐A2 (Hagino *et al*., 2013). The second group consisted of deeper samples (100, 125 and 140 m) dominated by a Pelagophyceae OTU (denovo167901), 100% identical to both *Pelagomonas calceolata* (Accession number LN735504) and the ‘wild *Pelagomonas*’ which is known to be a globally distributed open ocean uniflagellate (Worden *et al*., 2012). An additional *Braarudosphaera*‐related OTU (denovo55349; Fig. 2), with 97% identity to denovo283683, was detected in all samples with the highest relative abundances in samples from 125 and 140 m. Other classes of PPEs that were present in the sorts included *Dictyophyceae*, *Chrysophyceae*, *Bacillariophyta* and *Mamiellophyceae* (Fig. 2).

### Non‐chloroplast sequences in the PPE sorts

**Fig. 3:**
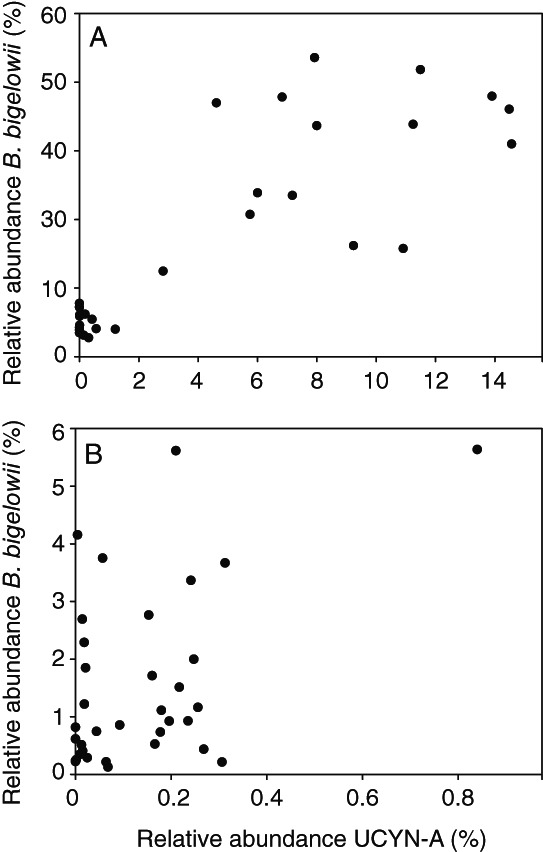
Scatter plots showing the relative abundance (%) of *B*. *bigelowii* (denovo283683) and UCYN‐A (denovo223459) sequences in A. PPE sorts and B. seawater samples.

To investigate bacterial cells co‐sorted with PPEs, the non‐chloroplast 16S rRNA gene OTUs present in the PPE sorts were explored. Potential associations between prokaryote and PPE cells could be identified as non‐chloroplast sequences that are enriched in the PPE sorts compared with the *Synechococcus* and *Prochlorococcus* population sorts which served as control populations. The most frequently detected non‐chloroplast sequence in the PPE sorts was that of UCYN‐A (denovo223459) (Accession number CP001842; 100% identity) which was up to 14.6% of the reads in a sample (Figs 1 and 3). The presence of UCYN‐A coincided with the dominance of *B*. *bigelowii* (denovo283683) sequences (Figs 2 and 3) and UCYN‐A sequences were largely absent in *Prochlorococcus* and *Synechococcus* sorts (Fig. 1). A *Prochlorococcus* affiliated OTU (denovo62755) was the second most prevalent non‐chloroplast sequence type with relative abundances of up to 1.8% in sorted PPE populations. *Synechococcus* and *Pelagibacter* OTUs were also present in the PPE sorts at maximum relative abundances of 0.4% and 0.5% respectively. However, no indications of enrichment of *Prochlorococcus*, *Synechococcus*, or *Pelagibacter* within the PPE sorts were observed as the relative abundances were similar or higher in the control sorts (Fig. 1). Thus, the presence of these phylotypes, widely present in seawater, is due to systematic background contamination due to sorting or sequencing biases such as demultiplexing errors rather than true associations within the PPE populations.

**Fig. 4:**
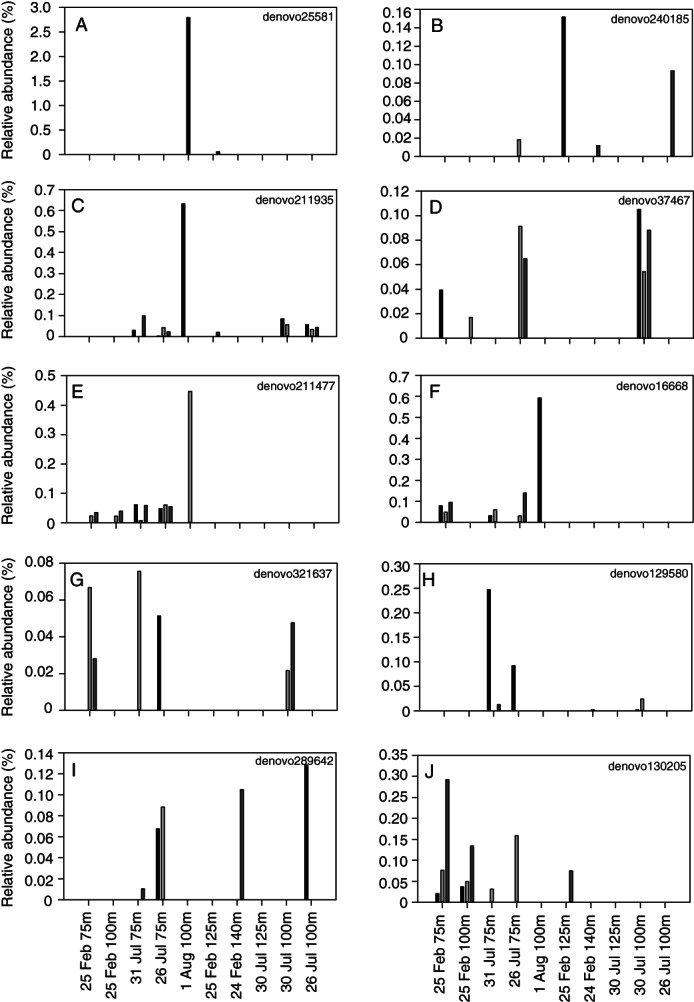
Bar graphs showing the relative abundance (%) of non‐chloroplast OTUs present in sorted PPE populations in triplicate sorts from each sample. A. *Hymenobacter*. B–F. ZB3. G–J. *Coxiellaceae*. Sample IDs correspond to PPE sorts ordered according to the composition of the chloroplast sequences in Fig. 1.

Groups of OTUs which could be of interest in terms of associations with the PPE populations were identified based on three criteria: (i) No representatives were present in negative extractions controls, sheath fluid controls, or negative PCR controls; (ii) There were no representatives in *Prochlorococcus* or *Synechococcus* control sorts; and (iii) There were greater than 100 sequences detected in PPE sort samples (representing >0.1% relative abundance in libraries). One group that met these criteria was represented by one OTU (denovo25581); a Bacteroidetes of the genus *Hymenobacter* which had 99% similarity to an uncultured bacterium from an Argentinian lake (Accession number KM142174). This OTU was present in two PPE sort samples at relative abundances of 2.8% and 0.1% respectively (Fig. 4A). A second group consisted of five OTUs related to the uncultivated bacterial candidate division ZB3 which was present at up to 0.6% of sequences in a sample but had a patchy distribution among replicates (Fig. 4B–F). A third group consisted of four OTUs related to the gammaproteobacterial family *Coxiellaceae* which was present at up to 0.3% of sequences in a sample (Fig. 4G–J).

### Characterization of single PPE sorts

In total, 388 single PPE cell genomes were amplified. The amplified samples were screened by amplification of 16S rRNA genes which resulted in 174 positive samples that were subsequently sequenced. After quality controls, including removal of contaminant sequences, 78 samples remained (Supplementary Table S4). The chloroplast sequences of the PPE cells were consistent with the genera present in the PPE population sorts (Fig. 2; Table S4). The most frequently detected OTUs present in the sorted single‐cell PPE samples, dominating in 39 of the samples, were those of UCYN‐A and/or its *Braarudosphaera* host (denovo223459 and denovo283683 respectively; Table S4). Both UCYN‐A and its host were recovered in several single‐cell sorts, but UCYN‐A was most frequently detected alone (18 samples in total, Table S4). Chloroplast OTUs which co‐occurred with the UCYN‐A host included OTUs affiliating with *Chrysochromulinaceae*, Prymnesiaceae, *Prymnesiophycidae*, *Phaeocystaceae*, *Camellia* and *Hymenomonadaceae*. Non‐chloroplast sequences present in the single PPE cell sorts included *Alteromonas macleodii*, *Pseudoalteromonas* sp., *Halomonas* sp. and *Idiomarina* species.

## Discussion

Knowledge about microbial associations is of high importance for understanding the role of interactions in ecological processes. Co‐occurrence analyses of organism abundance profiles can reveal interactions between organism groups within microbial assemblages (Lima‐Mendez *et al*., 2015). In recent years, FACS in combination with molecular identification at the single‐cell level has emerged as a tool for investigating associations between microorganisms (Martinez‐Garcia *et al*., 2012; Thompson *et al*., 2012; Bombar *et al*., 2013, 2014; Farnelid *et al*., 2016b; Brown *et al*., 2020). Because of the stringency used during flow cytometry sorting it is generally expected that only cells within the designated populations will be sampled and co‐sorted cells from other populations can be considered unlikely. The pressure in the fluidic lane will likely cause disruption of loosely associated cells but very tightly attached extracellular and intracellular cells will be co‐sorted with associated cells based on the properties (size and autofluorescence) of those cells. Although we anticipated finding multiple co‐sorted bacteria, our screening of PPE populations and single PPE cell sorts from the NPSG indicate that specific tight associations between prokaryotes and PPEs in the oligotrophic ocean appear to be unusual and that UCYN‐A and its host was predominant.

The N_2_‐fixing cyanobacterium UCYN‐A was the most frequently detected non‐chloroplast OTU in the sorted PPE populations, reaching the highest relative abundances (up to 14% of sequences) in the samples from 75 m and in two samples from 100 m (11589 and 11583). As identified by *nifH* amplicon sequencing and oligotyping analysis, UCYN‐A populations in the North Pacific Subtropical Gyre are typically dominated by the sublineage UCYN‐A1 followed by UCYN‐A3 (Turk‐Kubo *et al*., 2017). Since this study was based on 16S rRNA gene amplification, precluding sublineage identification, details of UCYN‐A sublineages are not discussed in this study. The occurrence of UCYN‐A sequences was correlated with the occurrence of chloroplast sequences of *B*. *bigelowii* (denovo286683; Figs 1, 2 and 3). Notably, this *B*. *bigelowii* OTU reached high relative abundances in some seawater samples also when UCYN‐A (denovo223459) had low relative abundance (Fig. 3). This is an indication that the distribution pattern of sequences related to *B*. *bigelowii* alone is not a reliable indicator of the presence and distribution of UCYN‐A. Among the single PPE cell sorts, nine samples contained sequences from both UCYN‐A and *B*. *bigelowii* (Table S4). Single PPE cell sort samples were sorted based on the properties of the host. Thus, the samples from which only *B*. *bigelowii* sequences were found (*N* = 5) could be examples of cells where the UCYN‐A has been dislodged from the association (Zehr *et al*., 2008; Tripp *et al*., 2010; Thompson *et al*., 2012) or a result of bias in genome coverage resulting from multiple displacement amplification (MDA) reactions. However, PPE cell samples where only UCYN‐A sequences were detected were more frequent (*N* = 18). The presence of UCYN‐A associated with its *B*. *bigelowii* host in both population and single PPE cell sorts serves as a positive control, illustrating that the methodology used in the current study was robust in identifying tight associations between bacterioplankton and PPEs.

Another reason that bacterial cells may be co‐sorted with PPE can be due to grazing. Multiple studies have suggested that PPEs may be mixotrophs, grazing on picocyanobacteria and heterotrophic bacterioplankton cells through bacterivory (Zubkov and Tarran, 2008; Frias‐Lopez *et al*., 2009; Sanders and Gast, 2012; Hartmann *et al*., 2013; McKie‐Krisberg and Sanders, 2014). In the oligotrophic ocean, where cell numbers are generally low, a random mode of grazing, based on encounter rates may be assumed. Hence, dominating taxa such as *Prochlorococcus* and the alphaproteobacterial SAR11 clade are likely to be the most abundant prey organisms (Fig. 2D). In the Atlantic Ocean, Hartmann *et al*. (2013) observed SAR11 and *Prochlorococcus* cells inside PPE (*Pelagophyceae*, *Chrysophyceae* and *Prymnesiophyceae*) cells. They also observed *Prochlorococcus* cells attached to the surface of PPE cells. *Prymnesiophyceae* cells showed internalization of *Prochlorococcus* at all studied stations (6% on average). Similarly, in the NPSG, Frias‐Lopez *et al*. (2009) identified phototrophic *Prymnesiophyceae* as grazers of both *Synechococcus* and *Prochlorococcus*, suggesting that these are key bacteriovores in the oligotrophic ocean. If PPEs contained ingested cells, and assuming that these were not already degraded, an enrichment of these taxa in the sorted PPE populations would be expected in this study. However, no systematic evidence of enrichments of either *Prochlorococcus*, SAR11, or *Synechococcus* could be linked to the sorted PPE populations or the single‐cell PPE sorts (Fig. 1; Table S4). This suggests that ingestion of these genera by mixotrophic PPE cells in the oligotrophic NPSG was limited at the time and location of our sampling.

Recently, Kamennaya *et al*. (2018) proposed the semi‐extracellular phagocytosis of *Prochlorococcus* cells by the prymnesiophyte *Braarudosphaera* which hosts UCYN‐A. Their identification of *Prochlorococcus* was solely based on the recovery of *Prochlorococcus* sequences in PPE sorts. In this study, using multiple control populations, we demonstrate that *Prochlorococcus* sequences could be detected but were not enriched with PPEs indicating a low level of DNA contamination of non‐PPE DNA in PPE sorts (Fig. 1). Such contamination likely led to the incorrect interpretation of the UCYN‐A symbiont as *Prochlorococcus* in electron microscopy images of *Braarudosphaera* stressing the importance of including control sorts when using FACS combined with molecular methods.

Three groups of prokaryotes were enriched in association with PPEs in the sorted populations (Fig. 4). These OTUs were affiliated with *Hymenobacter*, *Coxiellaceae* and the uncultivated bacterial candidate division ZB3. Members of the *Coxiellaceae* family are known as intracellular parasites and have been documented as obligate intracellular bacteria of marine amoeba (Schulz *et al*., 2015; Tsao *et al*., 2017) while ZB3 sequences have previously been identified in single‐cell sorts of Cercozoa and Chrysophyta (Martinez‐Garcia *et al*., 2012). The candidate division was first identified from a sulfide‐rich spring (Elshahed *et al*., 2003) but remains poorly characterized. Notably, the presence of these OTUs was patchy across replicate sorts and none of them was detected in the single‐cell sorts, suggesting that even if they are associated with PPEs, they are rare.

In this study, natural seawater samples were sorted within a short time after sampling (~3 h). The abundances of PPE cells in the NPSG are relatively low and therefore samples have traditionally been concentrated to allow for high‐throughput sampling. However, it has been found that the symbiosis between UCYN‐A and its host was easily disrupted during the concentration of samples (Thompson *et al*., 2012). While samples were treated as gently as possible in this study, it cannot be ruled out that loosely associated cells and even tight extracellular associations may have been disrupted during the sampling which would preclude detection of associated cells. Similarly, the size of the sorted populations (1000 PPE cells) may have been too few to identify extremely rare associations. Similarly, the results of the single‐sorted PPE cells were likely limited due to incomplete genome amplification in the MDA reaction (Mangot *et al*., 2017). However, the consistent detection of UCYN‐A in the PPE populations sorts and single‐cell sorts, representing a mere <0.2% of the sequences in the seawater samples (Fig. 1D) suggests that if specific tight associations were present, these could have been detected.

## Conclusion

In this study, we aimed to identify putative associations between prokaryotes and PPEs in the oligotrophic NPSG. The identification of species‐specific associations is a prerequisite for investigating their roles in microbial food webs and impacts on processing of organic matter and biogeochemical cycling dynamics. Through systematic screening of population sorts and single‐cell sorts, there was little evidence to support the presence of novel tight associations, suggesting that the known association between UCYN‐A and its host appears to be unique. It is possible that such associations may be more prevalent in eutrophic coastal environments or among other species of PPEs not represented in this study. However, demonstrating the rarity of such associations between marine prokaryotes and PPEs is of great importance for our understanding of interactions between organisms on the microscale. From an evolutionary perspective, symbiosis between single‐cell organisms has been fundamental in the evolution of complex, multicellular life (Zehr *et al*., 2017). Why only few examples of such consortia exist as of today remains an enigma.

## Experimental procedures

### Sampling

Seawater samples were collected during two cruises onboard the R/V Ka'imikai‐O‐Kanaloa (HOT269 and HOELegacy 2B cruise (HL2B)) in the NPSG in February and July 2015. Samples were collected from and around the DCM using a rosette of Niskin bottles equipped with a fluorometer and temperature, conductivity and oxygen sensors. For community DNA analyses, 2 L seawater samples were filtered gently through a 10 μm polycarbonate filter or a 3 μm polycarbonate filter and subsequently through a 0.2 μm Supor filter (Pall Corporation, New York, NY, USA) in 25 mm Swinnex filter holders using a peristaltic pump. The filters were placed in sterile 1.5 ml cryovials containing 0.1 g autoclaved glass beads, immediately flash‐frozen in liquid nitrogen and stored at −80°C until DNA extraction.

### Flow cytometry sorting

Populations and single cells of PPEs, *Prochlorococcus* and *Synechococcus* were sorted from fresh seawater samples using a BD Biosciences Influx Cell Sorter with a 488 nm laser (Sapphire Coherent) and a 86 μm diameter nozzle onboard the ship. BioSure Sheath fluid (BioSure, Grass Valley, CA, USA) at 1× concentration was prefiltered using a 0.22 μm Sterivex filter (Millipore). Sorting was triggered in the forward scatter (FSC) channel, using purity yield mode, and gates were constructed using FSC and chlorophyll *a* fluorescence with the Spigot software. Cell counts and cytograms were generated in FlowJo v10.0.7 (Tree Star, Ashland, OR, USA). Populations (1000 or 100 cells) or single PPE cells were sorted into sterile PCR tubes. Sheath fluid blanks were collected in parallel using a test deflection stream. Samples were stored at −80°C until further processing.

### DNA extraction

Community DNA was extracted using the Qiagen Plant Minikit (Qiagen) and partially automated using a QIAcube (Qiagen) with modifications as described below. The filters were thawed at room temperature and 400 μl AP1 buffer was added to the sample tubes. Thereafter the tubes were subjected to three freeze–thaw cycles using liquid nitrogen and a 65°C water bath (30s and 2 min respectively) followed by bead‐beating (FastPrep‐24 bead beater; MP Biomedicals, Irvine, CA, USA) at full speed for 2 min. The samples were Proteinase K treated for 1 h at 55°C with moderate shaking using 45 μl of Proteinase K (Qiagen, 20 mg ml^−1^) and then treated with 4 μl RNase A (Qiagen, 100 mg ml^−1^) and incubated for 10 min at 65°C. The filters were removed using sterile needles, 130 μl AP2 buffer was added to each tube, and samples were then vortexed and incubated on ice for 10 min. To pellet the precipitates and beads the tubes were centrifuged for 5 min at 18 800*g* at 4°C, in a Sorvall Legend Micro 21 (Thermo Electron Corporation) centrifuge and the supernatant was transferred to 2 ml sample tubes and placed in the QIAcube for further extraction steps according to the manufacturer's protocol. The samples were eluted using 100 μl AE buffer and concentrations were measured fluorometrically using Picogreen (ThermoFisher).

### Whole‐genome amplification from single cells

To amplify DNA from single PPE cells a modified MDA protocol was applied, which included additional steps of UV decontamination to reduce the risk of reagent contaminant amplification (Rinke *et al*., 2014). All handling of samples was done in a UV‐treated clean hood in a clean pre‐PCR room and for every strip (eight PCR tubes), a negative amplification control, i.e. a PCR tube without a sorted cell, was included. The amplification time was optimized to 3 h by observing the real‐time whole‐genome amplification in a qPCR instrument using test samples for different durations and assuring that negative controls were blank (Rinke *et al*., 2014). Reagents from the Qiagen REPLI‐g Single‐Cell Whole‐genome amplification kit were UV‐treated for 60 min prior to use. An amplification master mix was prepared by adding 4.5 μl H_2_O sc, 14.5 μl REPLI‐g sc Reaction Buffer and 1 μl REPLI‐g sc DNA polymerase per reaction. The mix was UV‐treated for 60 min and 0.5 μM SYTO13 was added to the mix. The cells were suspended in 2 μl PBS buffer and 1.5 μl Buffer D2 was added to each tube. The solution was mixed carefully and centrifuged briefly using a table centrifuge. The samples were incubated for 10 min in a 65°C water bath, 1.5 μl stop solution was added and the samples were stored on ice for <1 h. To each tube, 20 μl master mix was added and the whole‐genome amplification was observed in real‐time on a qPCR instrument set to 30°C for 3 h. The enzyme was inactivated for 3 min at 65°C. The whole‐genome amplified samples were diluted 1:100 and stored at −20°C until further analysis.

### PCR amplification and Illumina MiSeq sequencing of 16S rRNA genes

For all PCR preparations, extra precautions were taken to reduce the risks of contamination. PCR reagents and equipment were consistently UV‐treated before use and all preparations took place in a UV‐treated PCR hood in a clean pre‐PCR room using 5 kD filtered UV‐treated ultrapure water. Partial fragments of the 16S rRNA gene were amplified using the widely used primer set 341F and 806R (Caporaso *et al*., 2011), targeting the V3–V4 variable region. The amplification was done in a two‐step protocol as described by Green *et al*. (2015). The first PCR was performed in 25 μl reaction volumes with 0.25 μM of each primer containing CS1 and CS2 linkers (Moonsamy *et al*., 2013), 1× PCR buffer, 0.3 μl Invitrogen Platinum *Taq* (Invitrogen Carlsbad, CA, USA), 2.5 mM MgCl_2_ and 200 μM dNTP mix. The PCR conditions were 5 min initial denaturation at 95°C, annealing for 40 s at 53°C and elongation for 60 s at 72°C for 25 cycles and ended with a final elongation for 7 min at 72°C. For the extracted DNA samples, 2 μl solution was added to the PCR reactions as a template and for the sorted samples the PCR master mix was added directly into the sort tubes. Negative PCR controls with only ultrapure water added to the reactions and samples with sheath fluid that had passed through the flow cytometer were run together with the samples. The amplicons were submitted for sequencing to the DNA Services (DNAS) Facility at the University of Illinois at Chicago. There a second PCR reaction was conducted (8 cycles) incorporating barcodes (Fluidigm Access Array barcoding system) and sequencing adaptors into the final PCR products. The sequencing was performed on an Illumina MiSeq sequencer using V3 chemistry with paired‐end 300 base reads and demultiplexing of reads was performed on the instrument.

### Sequence analysis

The paired‐end FASTQ files were merged using the software package PEAR (Zhang *et al*., 2014). Quality filtering (phred20) and removal of putative chimera sequences was done using QIIME (Caporaso *et al*., 2010) and the usearch quality filtering pipeline (Edgar *et al*., 2011) with default parameters. OTU clusters (97% sequence similarity) were generated using the UCLUST method in the QIIME v1.8 pipeline (Caporaso *et al*., 2010) and singletons were removed. Sequences related to mitochondria and chloroplasts were identified using the Ribosomal Database Project (http://rdp.cme.msu.edu/) database and were separated from non‐Eukaryota related sequences. The chloroplast sequences were classified using NCBI blastn and the PhytoRef database for plastid 16S rRNA gene sequences (Decelle *et al*., 2015). The remaining sequences were taxonomically annotated using the SILVA rRNA gene database (Quast *et al*., 2013). For the libraries from single sorts, contaminant sequences were identified and removed from the libraries based on two criteria, (i) The OTU was only present in the samples from single sorts, i.e. not present in the population sort or seawater libraries, (ii) the closest relative was not associated with aquatic origin (this included sequences affiliating with *Streptococcus*, *Pasteurellaceae*, *Neisseriaceae*, *Veillonellaceae*, *Ruminococcaceae*, *Lachnospiraceae*, *Lactococcus*, *Granulicatella*, *Facklamia*, *Abiotrophia*, *Gemellaceae*, *Staphylococcus*, *Planococcaceae*, *Bacillus*, *Capnocytophaga*, *Prevotella*, *Porphyromonadaceae*, *Propionibacterium*, *Rothia*, *Microbacteriaceae*, *Dermabacter*, *Corynebacterium*, and *Actinomyces*; see Supplementary Tables S5 and S6). Samples with >1000 sequence reads and OTUs with >1% relative abundance were further analysed. The sequences have been submitted to NCBI Sequence Read Archive with accession number PRJNA608490.

## Supporting information


**Appendix S1:** Supporting information.

**Supplementary Table S2.** Summary of sequenced samples and sequence quality controls for size fractionated seawater samples.

**Supplementary Table S3.** Summary of sequenced samples and sequence quality controls for population sorts.

**Supplementary Table S4.** List of amplified genomes from single PPE sorts (total 78 out of 388) and the affiliations of 16S rRNA gene sequences divided into chloroplast and non‐chloroplast OTUs. For chloroplast sequences, the closest affiliation in PhytoRef is listed and for non‐chloroplast sequences the closest relative in NCBI (blastn) is listed by accession number in brackets. SAGs; Number of SAGs with the same chloroplast and non‐chloroplast composition. NA; No OTUs detected.

**Supplementary Table S5.** Summary of sequenced samples and sequence quality controls for single amplified genomes.

**Supplementary Table S6.** Summary of sequenced samples and sequence quality controls for negative control samples.
