# Supplementary material for: Cell sorting reveals few novel prokaryote and photosynthetic picoeukaryote associations in the oligotrophic ocean
Source: Environ Microbiol. 2020 Dec 19;23(3):1469–80. doi: 10.1111/1462-2920.15351 (PMC8048811; doi:10.1111/1462-2920.15351)

**Farnelid et al. Supplementary Figures and Tables**

**Supplementary Table S1.** List of samples, sampling depth, temperature and abundances of photosynthetic picoeukaryotes (PPE), *Prochlorococcus* (Pro), and *Synechococccus* (Syn) as measured by flow cytometry. NA; data not available.

| Cruise | Cast ID | Lat, Lon | Sampling Depth (m) | PPE (10^6^ cells L^-1^) | Pro (10^6^ cells L^-1^) | Syn (10^6^ cells L^-1^) |
| --- | --- | --- | --- | --- | --- | --- |
| HOT269 | S2C3 | 22.45, -157.59 | 140 | 1.6 | NA | 1.9 |
| HOT269 | S2C10 | 22.47, -157.60 | 100 | 2.0 | NA | 5.9 |
| HOT269 | S2C12 | 22.48, -158.40 | 75 | 1.8 | NA | 4.9 |
| HOT269 | S2C13 | 22.48, -158.21 | 125 | 1.5 | NA | 2.6 |
| HL2B | S6C1 | 24.49, -156.82 | 75 | 0.6 | 17.4 | 2.4 |
| HL2B | S6C1 | 24.40, -156.82 | 100 | 0.8 | 74.1 | 2.0 |
| HL2B | S12C1 | 24.67, -156.54 | 75 | NA | NA | NA |
| HL2B | S13C1 | 24.62, -156.46 | 75 | NA | NA | NA |
| HL2B | S14C1 | 24.61, -156.44 | 75 | NA | NA | NA |
| HL2B | S15C1 | 24.67, -156.41 | 75 | NA | NA | NA |
| HL2B | S17C1 | 24.58, -156.34 | 100 | 1.8 | 336.8 | 2.5 |
| HL2B | S17C1 | 24.58, -156.34 | 125 | 2.6 | 93.6 | 0 |
| HL2B | S22C1 | 24.51, -156.35 | 75 | 1.6 | 170.5 | 3.7 |
| HL2B | S25C1 | 24.49, -156.34 | 100 | 0.9 | 278.4 | 3.0 |

**Supplementary Table S2.** Summary of sequenced samples and sequence quality controls for size fractionated seawater samples.

**Supplementary Table S3.** Summary of sequenced samples and sequence quality controls for population sorts.

**Supplementary Table S4.** List of amplified genomes from single PPE sorts (total 78 out of 388) and the affiliations of 16S rRNA gene sequences divided into chloroplast and non-chloroplast OTUs. For chloroplast sequences, the closest affiliation in PhytoRef is listed and for non-chloroplast sequences the closest relative in NCBI (blastn) is listed by accession number in brackets. SAGs; Number of SAGs with the same chloroplast and non-chloroplast composition. NA; No OTUs detected.

**Supplementary Table S5.** Summary of sequenced samples and sequence quality controls for single amplified genomes.

**Supplementary Table S6.** Summary of sequenced samples and sequence quality controls for negative control samples.

*See tables in separate files.*

**Supplementary Figure S1:** Flow cytograms from samples collected during HOT269 corresponding to 100000 events. The cell populations *Prochlorococcus* (Pro), *Synechococcus* (Syn) and photosynthetic picoeukaryotes (PPE) were distinguished using red fluorescence as a proxy for chlorophyll *a* content, orange fluorescence as a proxy for phycoerythrin content and forward scatter (FSC) as a proxy for size. The abundances for the PPE populations (cells ml^-1^) for each sample is indicated in the cytograms. A)75 m, B) 100 m C) 125 m D) 140 m.


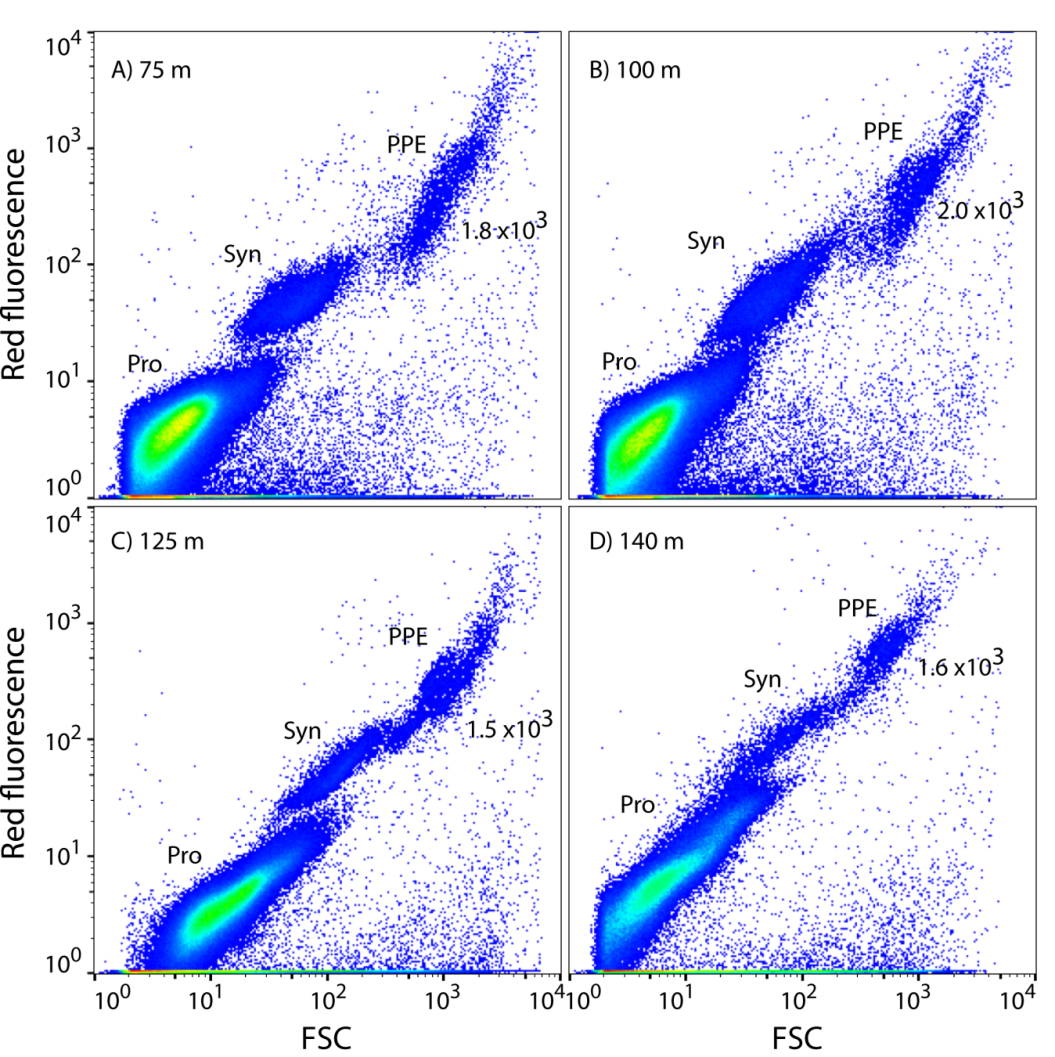


**Supplementary Figure S2:** Flow cytograms from samples collected during KOK1507 corresponding to 100000 events. The cell populations *Prochlorococcus* (Pro), *Synechococcus* (Syn) and photosynthetic picoeukaryotes (PPE) were distinguished using red fluorescence as a proxy for chlorophyll *a* content, orange fluorescence as a proxy for phycoerythrin content and forward scatter (FSC) as a proxy for size. The abundances for the PPE populations (cells ml^-1^) for each sample is indicated in the cytograms. A) S6C1 75 m, B) S6C1 100 m, C) S17C1 100 m, D) S17C1 125 m, E) S22C1 75 m, S25C1 100 m.


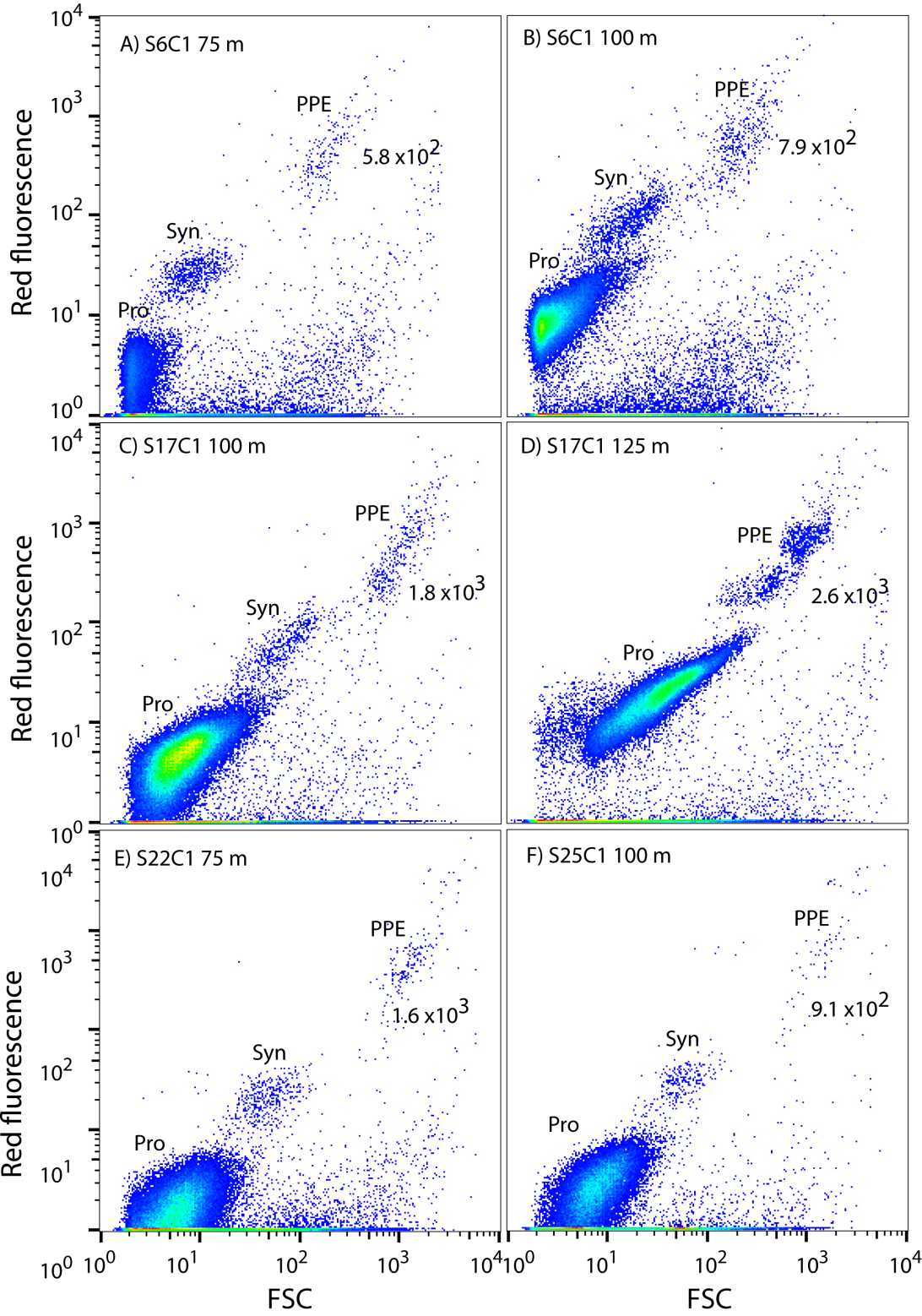

Supplement: Supplementary file 1 — Appendix S1: Supporting information. [file EMI-23-1469-s002.docx]
