# Supplementary material for: Cell sorting reveals few novel prokaryote and photosynthetic picoeukaryote associations in the oligotrophic ocean
Source: Environ Microbiol. 2020 Dec 19;23(3):1469–80. doi: 10.1111/1462-2920.15351 (PMC8048811; doi:10.1111/1462-2920.15351)
Supplement: Supplementary file 4 — Supplementary Table S4. List of amplified genomes from single PPE sorts (total 78 out of 388) and the affiliations of 16S rRNA gene sequences divided into chloroplast and non‐chloroplast OTUs. For chloroplast sequences, the closest affiliation in PhytoRef is listed and for non‐chloroplast sequences the closest relative in NCBI (blastn) is listed by accession number in brackets. SAGs; Number of SAGs with the same chloroplast and non‐chloroplast composition. NA; No OTUs detected. [file EMI-23-1469-s006.docx]

| SAGs | Chloroplast OTU identifier | Chloroplast OTUs | Non-Chloroplast OTU identifier | Non-chloroplast |
| --- | --- | --- | --- | --- |
| 1 | denovo283683; denovo349980 | 2 OTUs *Braarudosphaeraceae* (EU237456) | denovo223459 | Candidatus *Atelocyanobacterium thalassa (CP001842)* |
| 6 | denovo283683 | *Braarudosphaeraceae* (EU237456) | denovo223459 | Candidatus *Atelocyanobacterium thalassa* (CP001842) |
| 5 | denovo283683 | *Braarudosphaeraceae* (EU237456) | NA | NA |
| 1 | denovo283683 | *Braarudosphaeraceae* (EU237456) | denovo223459; denovo111312; denovo264342; denovo293688 | Candidatus *Atelocyanobacterium thalassa* (CP001842*); Pseudoalteromonas sp.* (MH158310); *Zunongwangia mangrovi* (NR_126281); *Alteromonas macleodii* (MH660284) |
| 1 | denovo283683 | *Braarudosphaeraceae* (EU237456) | denovo111312; denovo89855; denovo293688; denovo234368; denovo166492 | *Pseudoalteromonas* sp. (MH158310); *Halomonas* sp. (MG594829); *Alteromonas macleodii* (MH660284); *Idiomarina zobellii* (MH660323); Uncultured bacterium clone (JX227106) |
| 1 | denovo283683 | *Braarudosphaeraceae* (EU237456) | denovo223459; denovo111312 | Candidatus *Atelocyanobacterium thalassa (CP001842); Pseudoalteromonas sp. (MH158310)* |
| 1 | denovo283683; denovo225922; denovo208195; denovo25042 | *Braarudosphaeraceae* (EU237456); *Chrysochromulinaceae* (AY702175); *Chrysochromulinaceae* (EF574510); *Prymnesiaceae* (JX016584) | NA | NA |
| 2 | denovo283683; denovo25042; denovo225922; denovo246031 | *Braarudosphaeraceae* (EU237456); *Prymnesiaceae* (JX016584); *Chrysochromulinaceae* (AY702175); *Prymnesiophycidae* (HQ671857) | NA | NA |
| 1 | denovo283683; denovo25042; denovo208195; denovo55349; denovo225922; denovo246031; denovo238377; denovo223959 | *Braarudosphaeraceae* (EU237456); *Prymnesiaceae* (JX016584); *Chrysochromulinaceae* (EF574510); *Braarudosphaeraceae* (EF574745); *Chrysochromulinaceae* (AY702175); *Prymnesiophycidae* (HQ671857); *Chrysochromulinaceae* (EF573980); *Phaeocystaceae* (EU805127) | NA | NA |
| 1 | denovo283683; denovo25042; denovo208195; denovo225922; denovo246031; denovo55349 | *Braarudosphaeraceae* (EU237456); *Prymnesiaceae* (JX016584); *Chrysochromulinaceae* (EF574510); *Chrysochromulinaceae* (AY702175); *Prymnesiophycidae* (HQ671857); *Braarudosphaeraceae* (EF574745) | NA | NA |
| 1 | denovo283683; denovo25042; denovo343747 | *Braarudosphaeraceae* (EU237456); *Prymnesiaceae* (JX016584); *Chrysochromulinaceae* (HQ672034) | NA | NA |
| 1 | denovo56875 | *Braarudosphaeraceae* (GU061468) | denovo111312; denovo205396 | *Pseudoalteromonas* sp. (MH158310); Uncultured bacterium clone; Alphaproteobacteria, Rickettsiales (MG875982 ) |
| 1 | denovo56875; denovo247506; denovo276987 | *Braarudosphaeraceae* (GU061468); *Prymnesiophyceae* (GU940753); *Hymenomonadaceae* (GU940924) | NA | NA |
| 1 | denovo137158; denovo283683 | *Camellia* (NC_023084); *Braarudosphaeraceae* (EU237456) | NA | NA |
| 1 | denovo228711 | *Chromulinaceae* (EF520516) | denovo169144; denovo59520 | Uncultured bacterium clone; *Planctomycetes, Pirellulaceae* (KF786441); Uncultured bacterium clone; *Planctomycetes, Phycisphaerales* (KR857617) |
| 2 | denovo225922; denovo283683; denovo208195; denovo25042; denovo332050; denovo55349 | *Chrysochromulinaceae* (AY702175); *Braarudosphaeraceae* (EU237456); *Chrysochromulinaceae* (EF574510); *Prymnesiaceae* (JX016584); *Phaeocystaceae* (HQ163322); *Braarudosphaeraceae* (EF574745) | NA | NA |
| 1 | denovo156630 | *Chrysophyceae* (EF574962) | NA | NA |
| 1 | denovo150750 | *Chrysophyceae-Synurophyceae* (EF573915) | denovo288999 | *Fusobacterium periodonticum* (CP028108 ) |
| 1 | denovo286380; denovo116601; denovo345560; denovo74443; denovo274321; denovo127416 | *Dictyochophyceae* (AACY020080403); *Dictyochophyceae* (KC002426); 4 OTUs *Dictyochophyceae* (HQ671787) | NA | NA |
| 2 | denovo116601 | *Dictyochophyceae* (KC002426) | NA | NA |
| 1 | denovo128505 | *Dictyochophyceae* (KC002426) | NA | NA |
| 1 | denovo116601 | *Dictyochophyceae* (KC002426) | denovo111312 | *Pseudoalteromonas* sp. (MH158310) |
| 1 | denovo116601; denovo354484; denovo349168; denovo302948; denovo273570; denovo72501; denovo106122; denovo319229 | *Dictyochophyceae* (KC002426); 7 OTUs *Dictyochophyceae* (GQ250620) | NA | NA |
| 1 | denovo167901; denovo79435; denovo12539 | *Pelagophyceae* (HQ671892); 2 OTUs *Pelagophyceae* (EF574742) | denovo293688; denovo111312 | *Alteromonas macleodii* (MH660284)*; Pseudoalteromonas sp.* (MH158310) |
| 1 | denovo141629; denovo55349; denovo224805; denovo332050 | *Phaeocystaceae* (EF574075); *Braarudosphaeraceae* (EF574745); 2 OTUs *Phaeocystaceae* (HQ163322) | NA | NA |
| 1 | denovo99065 | *Prasinophyceae* (JX945378) | NA | NA |
| 1 | denovo180319 | *Prasinophyceae* (JX945378) | denovo111312 | *Pseudoalteromonas* sp. (MH158310) |
| 1 | denovo43947 | *Prymnesiaceae* (EF573693) | NA | NA |
| 1 | denovo246031; denovo283683; denovo225922 | *Prymnesiophycidae* (HQ671857); *Braarudosphaeraceae* (EU237456); *Chrysochromulinaceae* (AY702175) | NA | NA |
| 1 | NA | NA | denovo102035; denovo122811 | *Acinetobacter* sp. (MF495748 ); *Klebsiella oxytoca* (MF737172) |
| 2 | NA | NA | denovo293688 | *Alteromonas macleodii* (MH660284) |
| 18 | NA | NA | denovo223459 | Candidatus *Atelocyanobacterium thalassa* (CP001842) |
| 1 | NA | NA | denovo223459; denovo293688; denovo111312; denovo89855 | Candidatus *Atelocyanobacterium thalassa* (CP001842)*; Alteromonas macleodii* (MH660284)*; Pseudoalteromonas sp.* (MH158310); *Halomonas sp.* (MG594829) |
| 1 | NA | NA | denovo223459; denovo200021 | Candidatus *Atelocyanobacterium thalassa* (CP001842)*;* Uncultured bacterium clone; Deltaproteobacteria, OM27 (MF082316) |
| 1 | NA | NA | denovo167765 | *Dechloromonas agitata* (KM262801 ) |
| 1 | NA | NA | denovo89855 | *Halomonas* sp. (MG594829) |
| 1 | NA | NA | denovo89855; denovo2697; denovo223459 | *Halomonas* sp. (MG594829); *Idiomarina fontislapidosi* (MH588077); Candidatus *Atelocyanobacterium thalassa* (CP001842) |
| 5 | NA | NA | denovo111312 | *Pseudoalteromonas* sp. (MH158310) |
| 1 | NA | NA | denovo111312; denovo293688 | *Pseudoalteromonas sp.* (MH158310)*; Alteromonas macleodii* (MH660284) |
| 1 | NA | NA | denovo111312; denovo89855 | *Pseudoalteromonas* sp. (MH158310); *Halomonas* sp. (MG594829) |
| 1 | NA | NA | denovo260679 | Uncultured bacterium clone; Bacteroidetes, Flavobacteriaceae (JF272042) |
| 1 | NA | NA | denovo344537; denovo89855 | *Vibrio parahaemolyticus* (CP022244)*; Halomonas sp.* (MG594829) |
| 1 | NA | NA | denovo264342 | *Zunongwangia mangrovi* (NR_126281) |
| 1 | NA | NA | denovo264342; denovo234368 | *Zunongwangia mangrovi* (NR_126281)*; Idiomarina zobellii* (MH660323 ) |
